# A Review of Novel Antidepressants: A Guide for Clinicians

**DOI:** 10.7759/cureus.4185

**Published:** 2019-03-06

**Authors:** Amber E Faquih, Raheel I Memon, Hudaisa Hafeez, Muhammad Zeshan, Sadiq Naveed

**Affiliations:** 1 Psychiatry, Dow University of Health Sciences, Karachi, PAK; 2 Psychiatry, Henry Ford Allegiance Health, Jackson, USA; 3 Psychiatry, Duke University Health System, Durham, USA; 4 Psychiatry, Bronx Lebanon Hospital Icahn School of Medicine at Mount Sinai, Bronx, USA; 5 Psychiatry, Kansas University Medical Center, Kansas, USA

**Keywords:** antidepressant, snri, depression, selective serotonin reuptake inhibitors (ssri)

## Abstract

This review article aims to provide insight into the mechanisms of action, pharmacokinetics, clinical efficacy, safety and tolerability of four novel antidepressants including desvenlafaxine, vortioxetine, vilazodone, and levomilnacipran. Following keywords are used in PubMed and Scopus to search for relevant articles: (depression) AND (psychopharmacology OR desvenlafaxine OR levomilnacipran OR vortioxetine OR vilazodone). Patients with a lack of effectiveness or tolerability to certain antidepressants may get benefit from selecting a new antidepressant with different mechanism of action. These medications can be an option in the selection of newer antidepressants. Depression may not be caused by the simple deficiency of serotonin in the brain, but rather a complex interplay of various neurotransmitters including serotonin, norepinephrine, glutamate, and histamine at certain brain areas. The above-mentioned novel antidepressants exert their therapeutic benefits by acting on multiple neurotransmitters. The complexity of underlying the neurobiological mechanism should be considered while formulating a plan of care.

## Introduction and background

Major depressive disorder (MDD) is a major public health concern with significant impairment in psychological, occupational, and social functioning. The prevalence rates for depression are estimated to be around 3.2% in patients without comorbid physical illnesses and 9.3% to 23.0% in patients with chronic conditions. It is the fourth cause of disability around the world and is estimated to be the second leading cause of disability by 2020 [1]. It affects around 300 million individuals regardless of gender, ethnicity, geographical location, and socioeconomic status, contributing to the overall global burden of disease.

Selective serotonin reuptake inhibitors (SSRIs) are the appropriate first-line options for the treatment of depression along with psychotherapeutic interventions, but many patients either do not respond to different options or intolerant to the undesired effects of medications [2]. Despite multiple treatment regimen, about 60% of patients with MDD continue to report residual impairments even after treatment [3]. This residual symptoms and functional impairment have a higher risk of relapse into the future episodes of MDD. The debilitating health-related quality of life effects of MDD has an adverse impact on individuals, resulting in academic, interpersonal, social, and occupational impairment. Even after achieving remission, depression has higher rates of recurrence in up to 80% of all MDD patients with odds of becoming chronic in 20% of patients. The onset of each new major depressive episode increases the chances of relapse, chronicity, and treatment-resistant depression [4].

Different ideas have been postulated to understand the reasons for the ineffectiveness of monoamine modulators for the treatment of depression. The lack of effectiveness can result from poor compliance secondary to the delayed effects of the medications or undesired effects such as sexual dysfunction from the medications. It can be due to the severity of the depressive symptoms in patients struggling with treatment-resistant depression [5]. The guidelines recommend the selection of a different class of antidepressant with a different mode of action after the failure of antidepressant treatment with SSRIs or selective serotonin-norepinephrine reuptake inhibitor (SNRI). This recommendation is based on the fact that a medication with a different mechanism of action may have a better chance of success than the traditional antidepressants [3]. This recommendation is based on the heterogeneity of MDD in etiology, underlying the neurobiological mechanism, pathogenesis, course, and prognosis of illness. The serotonin and norepinephrine reuptake inhibitors have different potency of action at their respective receptors, resulting in distinctive clinical effects. With the distinctive treatment effect, the action of antidepressant medications, for example, SSRIs are restricted due to autoregulatory feedback mechanisms. To counteract the autoregulatory feedback, different methodologies are considered such as the addition of 5-HT_1A_,5-HT_1B_, and partial 5-HT_1A_ receptor agonism to SSRIs [6].

There are several newer treatment options including desvenlafaxine, vortioxetine, vilazodone, and levomilnacipran with antidepressant actions through different neurochemical actions. This review article educates the clinicians about the clinical factors including the mechanism of action, pharmacokinetics, clinical efficacy, and safety and tolerability. The authors also provide a summary of evidence-based studies regarding the newer antidepressants. This review articles explored the randomized controlled trials (RCTs), open-label trials, and case reports.

## Review

Review and search strategy

This article reviews the mechanism of action, pharmacokinetics, clinical efficacy, and safety and tolerability of desvenlafaxine, vortioxetine, vilazodone, and levomilnacipran extended release (ER). In April 2018, two electronic databases were systematically searched for relevant publications, including PubMed and Scopus, using the following search terms: (depression) AND (psychopharmacology OR desvenlafaxine OR levomilnacipran OR vortioxetine OR vilazodone). The manual search of references and relevant articles for included studies was also performed. Search results from these databases were imported to Endnote×7 (Thompson Reuter, CA, USA) to remove any duplicates. Two independent reviewers performed title and abstract screening (when available) followed by the full-text screening of 1674 included articles by selecting case reports, case series, open-label trials, and RCTs. In the case of disagreement, the consensus was reached by discussion among reviewers or guidance from a senior reviewer (SN). The abstract-only articles, conference papers without original data, review articles, theses, posters, book chapters, editorials, letters, commentaries were excluded. No restrictions on language, country, publication year, age, gender, or ethnicity of patients were applied. After the full-text screening, 41 studies were included in this review articles.

Desvenlafaxine

Desvenlafaxine, an active metabolite of venlafaxine, is an SNRI approved by the Food and Drug Administration (FDA) for the treatment of MDD in adults in 2009. It is administered as desvenlafaxine succinate [7]. 

Mechanism of action

Desvenlafaxine causes selective reuptake inhibition at the serotonin (5-HT) and norepinephrine (NE) transporters, resulting in an increased extracellular concentration of 5-HT and NE. The higher affinity for 5-HT transporters is observed compared to the NE transporters (approximately 10-fold) with a much weaker affinity for dopamine transporters (DATs). However, there are no functional implications on dopamine levels with the concentrations required to inhibit 5-HT and NE transporters for the antidepressant effect. Desvenlafaxine also affects hypothalamus which is an important regulator of biological functions like mood, sleep cycle, stress response, sexual behaviors, temperature, and pain sensations [8]. It lacks significant affinity for numerous receptors, including muscarinic cholinergic, histaminergic, and α1-adrenergic receptors, and monoamine oxidase (MAO) receptors [7], possibly reducing the risk of undesired side effects related to these receptor sites. 

Dosage

Desvenlafaxine is available in 50 and 100 mg tablets. The recommended dose for desvenlafaxine is 50 mg/day. In a patient with hepatic impairment, the dose above 100 mg/day is not recommended. In patients with moderate and severe renal impairment, the maximum dose was 50 mg/day and 50 mg every other day, respectively [7].

Pharmacokinetics

The bioavailability of desvenlafaxine is 80% after an oral administration and peak plasma concentration (Tmax) is seven and half hours after oral administration. It can be taken without meals as food does not appear to have a clinically significant difference. It is metabolized by conjugation and to a minor extent by oxidation through CYP3A4 [7]. Findling et. al reported a linear increase in Cmax and Area Under Curve (AUC) with an increase in the dose of desvenlafaxine in children and adolescents [9].

Clinical use

The efficacy of desvenlafaxine for MDD has been established in several RCTs and open-label studies among adults [9-17]. It improves the quality of life, as measured by an improvement in mood, social relationships, daily functioning, leisure activities, economic status, and physical mobility [13]. In patients with liver pathology, it alleviated the symptoms of depression [18-19]. In a case report of a patient with social phobia, the dose of 100 mg/day resulted in the remission of the symptoms [19]. An open-label trial also evaluated the efficacy of desvenlafaxine among children (aged 7-11 years) and adolescents (aged 12-17 years). The dose range of 50-200 mg was used in children (10-100 mg/day) and adolescents (25-200 mg/day) with a favorable response. The higher doses were associated with an increased incidence of treatment-emergent side effects [20]. Table 1 provides a summary of the characteristics of these trials including sample size, duration, doses, and age ranges of the participants. Table 2 summarizes the clinical outcomes for these studies. 

**Table 1:** Summary of demographic characteristics, dose ranges, and duration of studies of Desvenlafaxine A: Adolescent, C: Children, Case Report: CR, Fix: fixed dose, Flex: Flexible dose, OLT: Open-label trial, RCT: Randomized controlled trial

Studies	Design	Groups (n)	Duration (weeks)	Age (years)	Dose range (mg)
DeMartinis NA, et al., 2007 [11]	RCT	Desvenlafaxine 100 mg/day=114	8	18-75	100-400
		Desvenlafaxine 200 mg/day=116			
		Desvenlafaxine 400 mg/day=113			
		Placebo = 118			
Septien-Velez L, et al., 2007 [14]	RCT	Desvenlafaxine 200 mg=124	8	18-75	200-400
		Desvenlafaxine 100 mg= 125			
		Placebo=126			
Boyer P, et al., 2015 [15]	RCT	Desvenlafaxine 50 mg=166	8	>18	50-100
		Desvenlafaxine 100 mg= 148			
		Placebo=161			
Liebowitz MR, et al., 2008 [16]	RCT	Desvenlafaxine 50 mg=158	8	>18	50-100
		Desvenlafaxine 100 mg= 157			
		Placebo=159			
Feiger AD, et al., 2009 [6]	RCT	Desvenlafaxine = 117	8	>18	200-400
		Placebo = 118			
Dunlop BW, et al., 2011 [12]	RCT	Desvenlafaxine =285	12	18-75	50
		Placebo =142			
Clayton AD, et al., 2013 [10]	RCT	Desvenlafaxine = 217	8	40-70	50
		Placebo = 217			
Ferguson JM, et al., 2012 [13]	OL	104	12 months	18-75	200-400
Findling RL, et al., 2014 [20]	OL	Desvenlafaxine = 40	8 weeks (Fix)	7-17	10-100 (C)
		20 (C) and 20 (A)	6 months (Flex)		25-200 (A)
Findling RL, et al., 2016 [9]	OL	59	8	7-17	10-200
Fábregas B, et al., 2014 [18]	CR	2	8	45,49	100
Feinberg SS, et al., 2010 [19]	CR	1	12	27	100

**Table 2:** Summary of clinical outcomes of studies of Desvenlafaxine AUC: Area Under Curve, Des: Desvenlafaxine, IFN: Interferon, MDD: Major Depressive Disorder

Studies	Clinical outcomes
DeMartinis NA, et al., 2007 [11]	Des 100 and 400 mg helped with depression, 200 mg was not statistically significant compared to placebo.
Septien-Velez L, et al., 2007 [14]	Des 200 and 400 mg was effective compared to placebo for depressive symptoms and global functioning.
Boyer P, et al., 2015 [15]	Des 50 and 100 mg was effective compared to placebo for depressive symptoms and global functioning.
Liebowitz MR, et al., 2008 [16]	Des 50 and 100 mg was effective for depression, 100 mg was not statistically significant compared to placebo.
Feiger AD, et al., 2009 [6]	Des 200 and 400 mg was effective compared to placebo for depression but failed to reach statistical significance.
Dunlop BW, et al., 2011 [12]	Response and remission rates were 60% and 42% with des compared to 42% and 30% with placebo.
Clayton AD, et al., 2013 [10]	Des helped with depression and functional status among perimenopausal and post-menopausal women.
Ferguson JM, et al., 2012 [13]	High dose des was found to be safe and effective for MDD for up to a year.
Findling RL, et al., 2014 [20]	Des was effective for depression in short-term and long-term duration.
Findling RL, et al., 2016 [9]	Des was safe and well-tolerated in youth. The AUC was found to be linear with dosage.
Fábregas B, et al., 2014 [18]	Des helped with depression with full remission after IFN in one patient. In second patient, there was only minor improvement.
Feinberg SS, et al., 2010 [19]	Des 100 mg was effective for social phobia in a patient with Gilbert syndrome.

Safety and tolerability

The treatment-emergent adverse effects (AEs) are dose-related with desvenlafaxine and are the most common reason to discontinue treatment. Serious AEs were reported in 0.9% of patients taking desvenlafaxine compared to 0.4% taking a placebo. An analysis of AEs reported completed suicide in one, suicidal attempt in three, and suicidal ideations in five patients taking desvenlafaxine. However, the difference in suicidal behaviors was not statistically significant among desvenlafaxine and placebo groups. The most common AEs are nausea, headache, dizziness, insomnia, and dry mouth. Other side effects included fatigue, hyperhidrosis, tremors, blurred vision, sedation, increased anxiety, mydriasis, dyspepsia, affective lability, muscle spasms, and taste perversion. In patients taking desvenlafaxine, vital signs, laboratory results, and electrocardiogram (EKG) findings were clinically insignificant. Small increases in pulse and blood pressures were observed possibly due to noradrenergic effects of desvenlafaxine but they were clinically insignificant. Desvenlafaxine was associated with a lower risk of QTc prolongation compared to placebo. Dose-dependent lipid abnormalities were clinically significant in 1% of patients [21]. Abrupt discontinuation or dose reduction were associated with dizziness, nausea, headache, irritability, insomnia, diarrhea, anxiety, fatigue, abnormal dreams, and hyperhidrosis. The discontinuation rates are higher among patients taking it for a longer duration, needing a gradual taper [7].

Pregnancy

In pregnant rats and rabbits, desvenlafaxine causes a decrease in fetal weight with no evidence of teratogenicity. However, there are not adequate studies in pregnant women [7].

Vortioxetine

Vortioxetine was approved for the treatment of MDD in 2013 by the FDA. It contains the beta polymorph of vortioxetine hydrobromide and administered as an immediate-release tablet [22]. 

Mechanism of action

Vortioxetine exerts its therapeutic effects through multimodal activity with a modulation of the 5-HT receptors and an inhibition of the 5-HT transporters [22]. The antidepressant effect of the vortioxetine is mediated through its antagonist effect on the 5-HT_3_, 5-HT_7_, and 5-HT_1D_ receptors, partial agonist activity on the 5-HT1B receptor, agonist effect on the 5-HT1A receptor, and inhibition of the 5-HT transporter. Beside the modulatory effect on the 5-HT receptors and transporter, it also enhances the extracellular concentration of various neurotransmitters such as dopamine, histamine, noradrenaline, and acetylcholine [23].

At the standard dose of SSRI and SNRI medications, 80% occupancy of the serotonin transporter (SERT) is achieved, however 5 mg of vortioxetine occupies about 40% of the SERT suggesting the role of other pharmacological actions. In the healthy participants, the serotonin occupancy of vortioxetine was ≈ 50% at 5 mg, 65% at 10 mg, and >80% at 20 mg. The series of inhibitory feedback in response to prolonged SERT blockade decreases the extracellular 5-HT concentration and also attenuates the activation of 5-HT_1A_ and 5-HT_1B_ auto-receptors of the serotonergic system. The partial agonist activity of vortioxetine counteracts the 5-HT1B autoreceptor activity and in turn, increases the serotonin synthesis and release. The full agonist activity of vortioxetine on the 5HT_1A_ receptor normalizes the serotonin release through the likely mechanism of rapid desensitization of 5HT_1A_ auto-receptors. The antagonist effect on the 5-HT_3_ receptor inhibits the GABA-mediated inhibition of the interneurons, thereby increasing the extracellular serotonin concentration. Similarly, the blockade of 5-HT_7_ and 5HT_1D_ receptors has shown the effect of antidepressants in an animal study. The 5-HT system has a favorable impact on mood, affect, and cognition. Vortioxetine modulates the essential neurotransmitters which are involved in cognitive regulation such as glutamate, acetylcholine, histamine, dopamine, and noradrenaline [24].

Dosage

Vortioxetine is available as 5, 10, and 20 mg tablets. It can be started at 10 mg once daily and the dose can be increased to 20 mg based on the tolerability and clinical response [22].

Pharmacokinetics

In vivo, vortioxetine shows a linear, and dose-proportional relationship, i.e. c-max, and AUC increases linearly with dose (2.5-60 mg). It has moderate oral bioavailability that is independent of food intake. Vortioxetine has an extensive volume of distribution as the plasma protein binding is 80-90%. The study also revealed a significant separation of vortioxetine from placebo over the graph at the higher dose (20 mg) suggesting that a higher dose may potentially yield a better outcome. It takes 3-16 hours to attain maximum plasma concentration after dosing, and with multiple doses, the terminal half-life is approximately 60-70 hours. Vortioxetine is metabolized extensively in the liver and is the substrate for numerous CYP450 isoforms, primarily CYP2D6. In the liver, the CYP450 isoforms extensively metabolize the vortioxetine through oxidation and subsequent glucuronic acid conjugation to a pharmacologically inactive carboxylic acid metabolite. The medication itself does not have an inhibitory or excitatory effect on CYP1A2, CYP2C9, CYP2C19, CYP2D6 or CYP3A4 [24-25]. However, the increased risk of bleeding is explained by inhibition of serotonin uptake by platelets, an increase in gastric acid secretion, and modulation of the CYP450 metabolism [26].

Clinical use

The efficacy of vortioxetine for MDD was established in nine RCTs in adults at a dose range of 5-20 mg [3, 25, 27-33]. The doses of 5-20 mg were found to be in effective range with a lack of effectiveness with lower doses of 2.5-5 mg [24]. The maximum dose of vortioxetine 20 mg exhibits a greater clinical response compared to the lower doses for vortioxetine [27-30]. This dose of 20 mg was also associated with a higher risk of side effects. The improvement in global functioning [23,25,28-29,31-33], quality of life [28-29,32], and anxiety [23,25,28-29, 31-32,34] was also reported with vortioxetine. In an open-label trial of 52 weeks, it was effective in relapse prevention of depressive symptoms [34]. Table 3 provides a summary of the characteristics of these trials including sample size, duration, doses, and age ranges of the participants. Table 4 summarizes the clinical outcomes for these studies. 

**Table 3:** Summary of demographic characteristics, dose ranges, and duration of studies of Vortioxetine Lu:LuA21004, No.: number, OLT: Open-label trial, RCT: Randomized controlled trial, XR: Extended release

Studies	Design	Groups (n)	Duration (weeks)	Age (years)	Dose range (mg)
Alvarez E, et al., 2012 [25]	RCT	Lu 5 mg=109	6	18-65	Lu=5, 10
		Lu 10 mg=101			
		Placebo=105			Venlafaxine=225
		Venlafaxine XR=114			
Baldwin DS, et al., 2012 [23]	RCT	Lu 2.5 mg=155	8	18-75	Lu=2.5, 5, 10
		Lu 5 mg=159			
		Lu 10 mg=153			Duloxetine=60
		Duloxetine=157			
		Placebo=152			
Boulenger JP, et al., 2012 [30]	Phase1=OLT	OLT=639	OLT=12 weeks	18-75	5, 10
	Phase 2=RCT	RCT: Lu=204			
		Placebo=192	RCT=26-64 weeks		
Jain R, et al., 2013 [31]	RCT	Vortioxetine 5 mg=300	6	18-75	5
		Placebo=300			
Mahableshwarkar AR, et al., 2013 [33]	RCT	Vortioxetine 2.5 mg=153	8	18-75	2.5, 5
		Vortioxetine 5 mg=153			
		Placebo=300			
Boulenger JP, et al., 2014 [27]	RCT	Vortioxetine 15 mg=152	8	18-75	Vortioxetine=15,20
		Vortioxetine 20 mg=151			
		Duloxetine=147			Duloxetine=60
		Placebo=158			
Montgomery SA, et al., 2014 [3]	RCT	Vortioxetine 5 mg=255	12	18-75	10-20
		Agomelatine=246			
Chen G, et al., 2015 [26]	RCT	Aspirin and vortioxetine	20 days	18-45	Venlafaxine=10
		Warfarn and vortioxetine	2 weeks		
Jacobsen PL, et al., 2015 [28]	RCT	Vortioxetine 10 mg=155	8	18-75	10-20
		Vortioxetine 20 mg=150			
		Placebo=157			
Mahableshwarkar AR, et al., 2015 [29]	RCT	Vortioxetine 15 mg=147	6	18-75	Vortioxetine=15,20
		Vortioxetine 20 mg=154			
		Duloxetine=152			Duloxetine=60
		Placebo=161			
Alam MY, et al., 2014 [34]	OLT	310	52	Mean age=45.5	2.5-10

**Table 4:** Summary of clinical outcomes of studies of Vortioxetine Lu=LuA21004

Studies	Clinical outcomes
Alvarez E, et al., 2012 [25]	Lu helped with depression, anxiety, and global functioning compared to placebo.
Baldwin DS, et al., 2012 [23]	Lu and duloxetine helped with depression but it failed to reach statistical significance compared to placebo.
Boulenger JP, et al., 2012 [30]	Lu helped with relapse of depression compared to placebo.
Jain R, et al., 2013 [31]	The was no significant difference in symptoms of depression at week 6 compared to placebo.
Mahableshwarkar AR, et al., 2013 [33]	Vortioxetine 2.5 and 5 mg helped with depression compared to placebo but it was not statistically significant.
Boulenger JP, et al., 2014 [27]	Lu and duloxetine resulted in an improvement in depression and anxiety compared to placebo.
Montgomery SA, et al., 2014 [3]	Vortioxetine was significantly superior to placebo for depression, anxiety and global functioning.
Chen G, et al., 2015 [26]	Vortioxetine had no effect on the steady-state pharmacokinetics of aspirin and warfarin.
Jacobsen PL, et al., 2015 [28]	Vortioxetine 20 mg was significantly superior to placebo for depression compared to placebo.
Mahableshwarkar AR, et al., 2015 [29]	Vortioxetine 20 mg helped with depression and anxiety.
Alam MY, et al., 2014 [34]	Participants taking vortioxetine experienced improvement in depression and anxiety symptoms.

Safety and tolerability

The side effect profile of vortioxetine has an indirect effect on the management of depressive symptoms. These side effects result in the discontinuation of vortioxetine (3-11%) compared to placebo 1-8% [23,27]. In two studies of vortioxetine, nausea was the most frequent AE with an incidence reported as 10% [28-29]. 

The most common AEs reported in at least 5% of patients in order were nausea, headache, diarrhea, and dry mouth [28]. Sexual dysfunction is not an uncommon side effect of medication with serotonin reuptake inhibitor and is one of the reasons for treatment discontinuation and noncompliance [29]. Interestingly, the side effects including abnormal orgasm, ejaculation disorder, decreased libido, erectile dysfunction were reported to be higher with a low dose of vortioxetine 2.5 mg compared to 5 mg dose [33]. The gender has a variable effect on the incidence of AEs in vortioxetine with men at a higher risk compared to women [23].

The incidence of hemorrhage in a short-term clinical study with a dose range of 10 -20 mg/day was lower, and it was similar between vortioxetine (1.7%) and placebo (1.2%) participants [26]. In a study of vortioxetine 15 mg, two serious AEs (stress fracture and suicidal ideation) were reported [29]. There was no clinically significant relevance to suicidal ideation and behavior as measured by the Columbia Suicide Severity Rating Scale [27,29,33]. There was no change in vital signs, laboratory test results, weight, or EKG parameters for participants [23,25,29-30]. Sudden discontinuation of vortioxetine after two weeks of treatment showed no clinically significant withdrawal effect versus placebo [28-29].

Pregnancy

In a pregnant patient, vortioxetine 5 mg was discontinued after one month. Delivery of the healthy baby (3800 mg in weight) was reported [23]. It can cause decreased fetal weight and delayed ossification in animal studies [22].

Vilazodone

Vilazodone, an SSRI and a partial 5-HT_1A _receptor agonist, was approved for the treatment of MDD in adults by FDA in 2011 [35]. 

Mechanism of action

Vilazodone enhances serotonergic activity by selective inhibition of serotonin reuptake through the blockade of SERT. This effect is further intensified because of partial agonist activity at 5-HT_1A_ receptors [35].

Dosage

Vilazodone tablets are available in 10,20, and 40 mg strengths. The recommended dose range is 20-40 mg once daily. Vilazodone should be titrated, starting with an initial dosage of 10 mg once daily for seven days which can be titrated to 20 mg once daily. The dose can be increased to 40 mg once daily after seven days. The administration of Vilazodone without food affects the systemic bioavailability and diminished clinical effectiveness due to inadequate drug levels [35].

Pharmacokinetics

Vilazodone has a dose-proportional pharmacokinetic activity (5-80 mg). It reaches peak concentration in four to five hours after administration and has an elimination half-life of 25 hours. It is widely distributed and is 96-99% protein bound. Vilazodone is extensively metabolized through both cytochrome P450 (CYP) pathways (major: CYP3A4, minor: CYP2C19 and CYP2D6), with little to unchanged drug recovered in the urine (1%) and feces (2%) [35].

A sequence of open-label studies conducted by Boepally and colleagues identified the effect of CYP3A4 inhibition or induction on the pharmacokinetics of vilazodone. The average AUC of vilazodone exposure increased from 42-51% if given with ketoconazole, however, the co-administration of vilazodone and carbamazepine XR, a CYP3A4 inducer, decreased mean steady-state vilazodone exposure by 45%. The results suggest that up to a 50% decrease of vilazodone dosage should be considered when it is given in combination with strong CYP3A4 inhibitors; conversely, increasing the vilazodone dosage up to a maximum of 80 mg/day should be considered when it is given in combination with strong CYP3A4 inducers [36].

Clinical use

The efficacy of vilazodone in the treatment of MDD was established in two RCTs [37-38]. The participants in these studies reported an improvement in depressive symptomatology including depression-related anxiety symptoms and overall disease severity. These findings from these pivotal studies were replicated in another study with randomization to 40 mg/day of vilazodone or placebo for eight weeks of a double-blind trial [39]. Vilazodone resulted in significant improvements in their symptoms of depression and anxiety compared to placebo from the baseline to endpoint. Vilazodone 40 mg is noted to be an effective treatment choice in depression and anxiety with a resultant decrease in scores on the corresponding scales. In a study, 79 adults with MDD were enrolled in an open-label study of citalopram 20 mg/day for six weeks [40]. The symptomatic patients after a six-week trial of citalopram 20 mg were randomly assigned to either higher dose of citalopram 40 mg/day or to a vilazodone 40 mg/day. Both groups showed decreases in all outcome measures, but there were no significant differences between groups. Initial non-responders to a low dose of citalopram seem equally likely to respond to a higher dose of citalopram or to vilazodone. These findings were replicated in another study comparing the efficacy of citalopram and vilazodone [2]. Table 5 provides a summary of the characteristics of these trials including sample size, duration, doses, and age ranges of the participants. Table 6 summarizes clinical outcomes for these studies. 

**Table 5:** Summary of demographic characteristics, dose ranges, and duration of studies of Vilazodone DB: Double-blinded, RCT: Randomized controlled trial

Studies	Design	Groups (n)	Duration (weeks)	Age (years)	Dose range (mg)
Rickels K, et al., 2009 [37]	RCT	Vilazodone=198	8	18-65	40
		Placebo=199			
Khan A, et al., 2011 [38]	RCT	481	8	18-70	40
Boinpally R, et al., 2014 [36]	RCT	Vilazodone and Ketoconazole=37	8	18-60	5-10
		Vilazodone and Carbamazepine=30			40
Croft HA, et al., 2015 [39]	RCT	Vilazodone=253	8	18-70	50
		Placebo=252			
Matthews M, et al., 2015 [2]	RCT	Vilazodone 20 mg=292	Single dose	32-76	
		Vilazodone 40 mg=291	10	18-70	20-40
		Citalopram 40 mg=289			
		Placebo=290			
Rele S, et al., 2015 [41]	RCT	Vilazodone 10 mg=20	8	18-65	10-40
		Vilazodone 20 mg=20			
		Vilazodone 40 mg=20			
Grant JE, et al., 2017 [40]	RCT	Study 1: Citalopram 20 mg=79	12	18-60	40
		Study 2: Citalopram 40 mg=23			
		Study 2: Vilazodone 40 mg=19			

**Table 6:** Summary of clinical outcomes of studies of Vilazodone

Studies	Clinical outcomes
Rickels K, et al., 2009 [37]	Vilazodone-treated patient had higher response compared to placebo with improvement.
Khan A, et al., 2011 [38]	Vilazodone resulted in significant improvement in depressive symptoms compared to placebo
Boinpally R, et al., 2014 [36]	50% dose reduction of vilazodone is needed with strong CYP3A4 inhibitors.
	Vilazodone dosage up to a maximum of 80 mg with strong CYP3A4 inducers.
Croft HA, et al., 2015 [39]	Statistically significant outcomes were found for vilazodone 40 mg/day versus placebo.
Matthews M, et al., 2015 [2]	Vilazodone 20/40 mg was equal in efficacy and tolerability as citalopram 40 mg compared to placebo.
Rele S, et al., 2015 [41]	There was a significant improvement in depression measures from baseline to the end of the study.
Grant JE, et al., 2017 [40]	Initial non-responders to a low dose of citalopram seems equally likely to respond to a higher dose of citalopram or to vilazodone.

Safety and tolerability

The discontinuation rates due to AEs were higher with vilazodone than placebo [41]. The most common AEs of vilazodone were vomiting, nausea, diarrhea, insomnia, somnolence, dizziness, and dry mouth [35]. There were no noticeable changes in vital signs, in laboratory test results, weight, or EKG parameters among different treatment groups [35]. There was no clinically significant relevance to suicidal ideations and behaviors in short-term studies [2].

In a study, the safety of vilazodone was compared to placebo, and citalopram was included as an active control. Both vilazodone and citalopram were generally well tolerated [39]. The most commonly reported AEs leading to discontinuation were nausea (placebo, n =1; vilazodone 20 mg/day, n =6; vilazodone 40 mg/day, n =3, citalopram, n= 4). The incidence of vomiting and somnolence was greater in the vilazodone 40 mg/day group compared to the vilazodone 20 mg/day group. Overall, the incidence of AEs related to sexual functioning was higher in the active treatment groups relative to placebo and they were more frequent in the citalopram group. No patient in the vilazodone groups withdrew due to sexual side effects. Abrupt discontinuation or dose reduction lead to dysphoric mood, irritability, agitation, dizziness, sensory disturbances (e.g., paresthesia, such as electric shock sensations), anxiety, confusion, headache, lethargy, emotional lability, insomnia, hypomania, tinnitus, and seizures [35].

Pregnancy

Vilazodone is reported to cause developmental toxicity in rats but teratogenicity has not been reported in animal studies. There is a lack of adequate and well-controlled studies of vilazodone in pregnant women, needing a careful analysis of potential benefits and risks of treatment [35].

Levomilnacipran ER

Levomilnacipran ER, 1S, 2R- milnacipran, is an SNRI approved by the FDA for the treatment of MDD in adults [42].

Mechanism of action

All SNRIs inhibit the reuptake of 5-HT and NE with a difference in their potencies for the respective transporters, resulting in clinical implications [43]. Levomilnacipran ER is a relatively more active enantiomer of racemic milnacipran. It has two-folds higher potency for norepinephrine compared to serotonin reuptake inhibition and preferentially inhibits norepinephrine reuptake compared to duloxetine, venlafaxine, and desvenlafaxine [44]. It lacks effect on other receptors, ion channels or transporters tested in vitro, including serotonergic (5HT_1-7_), α- and β- adrenergic, muscarinic, or histaminergic receptors and Ca2+, Na+, K+ or Cl- channels [45].

Dosage

Levomilnacipran is an ER formulation, allowing once-daily dosing. The recommended dose range for levomilnacipran is 40-120 mg/day. The starting dose is 20 mg /day and then it can be increased to 40 mg/day after two days. The dose can be further titrated in increments of 40 mg every two or more days [45].

Pharmacokinetics

Levomilnacipran ER is well absorbed after oral administration with a bioavailability of 92%. The mean time to achieve peak plasma concentration is six to eight hours after oral administration and has a protein binding of 22%. Food does not affect the oral bioavailability of levomilnacipran. It is metabolized by CYP3A4 with minor involvement of other pathways in the CYP450 system [45]. It has an elimination half-life of around 12 hours with a prolongation in patients with renal impairment [46].

Clinical use

The efficacy of levomilnacipran for MDD and global functioning is established in five RCTs among adults at doses of 40-120 mg/day [44,47-50]. In an RCT by Gommoll et. al, there was an improvement in depressive symptoms, but it didn’t reach statistical significance [50]. Asnis et al. (2013) suggested a superior response for 80 mg and 120 mg doses compared to 40 mg [47]. Table 7 provides a summary of the characteristics of these trials including sample size, duration, doses, and age ranges of the participants. Table 8 summarizes the clinical outcomes and findings from these studies. 

**Table 7:** Summary of demographic characteristics, dose ranges, and duration of studies of Levomilnacipran ER ER: Extended release, OLT: Open-label trial, RCT: Randomized controlled trial, RF: Renal functioning

Studies	Design	Groups (n)	Duration (weeks)	Age (years)	Dose range (mg)
Montgomery SA, et al., 2013 [48]	RCT	Levomilnacipran ER=282	10	18-70	75-100
		Placebo=281			
Asnis GM, et al., 2013 [47]	RCT	Levomilnacipran ER 40 mg=181	11	18-65	40-120
		Levomilnacipran ER 80 mg=181			
		Levomilnacipran ER 120 mg=183			
		Placebo=179			
Bakish D, et al., 2014 [44]	RCT	Levomilnacipran ER 40 mg=185	10	18-75	40-80
		Levomilnacipran ER 80 mg=187			
		Placebo=185			
Grommoll CP, et al., 2014 [50]	RCT	Levomilnacipran ER=175	8	18-80	40-120
		Placebo=182			
Sambunaris A, et al., 2014 [49]	RCT	Levomilnacipran ER=222	8	18-80	40-120
		Placebo=220			
Chen L, et al., 2015 [42]	OLT	32 in four groups based on RF	Single dose	32-76	
Chen L, et al., 2015 [46]	OLT	Levomilnacipran ER and Ketoconazole=34	Variable	18-45	40-120
		Levomilnacipran ER and Carbamezapine=34			
		Levomilnacipran ER and Alprazolam=30			

**Table 8:** Summary of clinical outcomes of studies of Levomilnacipran ER ER: Extended release, No.: Number, RCT: Randomized controlled trial

Studies	Clinical outcomes
Montgomery SA, et al., 2013 [48]	Levomilnacipran ER had a favorable response for depression and functional impairment.
Asnis GM, et al., 2013 [47]	Significant improvement in depression at 80 and 120-mg doses than in the 40-mg group. All doses were superior to placebo.
Bakish D, et al., 2014 [44]	Levomilnacipran ER had a clinically significant improvement in depression and functional impairment for 40 and 80 mg doses.
Grommoll CP, et al., 2014 [50]	Levomilnacipran ER resulted in a greater reduction in depressive symptoms but was not statistically significant from placebo.
Sambunaris A, et al., 2014 [49]	Levomilnacipran ER resulted in improvement of the depressive symptoms and functional impairment.
Chen L, et al., 2015 [42]	No dose adjustment is needed with mild renal impairment but need adjustments with moderate and severe impairment.
Chen L, et al., 2015 [46]	Dose reduction with ketoconazole and dose adjustment is needed with CYP3A4 inducers or alprazolam.

Safety and tolerability

The safety of levomilnacipran SR for various dosages of 40, 80, and 120 mg was assessed in an RCT. The most common treatment-emergent AEs, defined as ≥ 10% of any treatment group, were headache, nausea, constipation, dry mouth, increased heart rate, and hyperhidrosis. During double-blind treatment, serious AEs were reported in two patients (1.1%) in the levomilnacipran ER 40-mg group (chest pain and deep vein thrombosis in one patient, and aggression in one patient) and one patient (0.6%) in the 80-mg group (cytomegalovirus mononucleosis). During medication taper, a new AE emerged; Nasopharyngitis which was more frequently reported in 40 mg group (zero patients in the placebo group; three patients in the 40-mg group; one each in the 80-mg and 120-mg groups). Other AEs included increases in liver transaminases and a mild increase in blood pressure. Similar trends of AEs were reported in another RCT of two dosages 40 and 80 mg [44]. Two patients in the levomilnacipran ER 40 mg/day group exited the study owing to serious AEs (intussusception, n= 1, and asthma, n= 1). No serious AEs were reported in the levomilnacipran ER 80 mg/day group, and no serious AE was considered by the investigator to be treatment-related [47]. The AEs that occurred at an incidence of 5% or more and at least at twice the rate of placebo in both levomilnacipran ER groups were same as Asnis et al. but also included urinary hesitation and erectile dysfunction.

Pregnancy

Levomilnacipran lacks adequate and well-controlled studies in pregnant women. Neonates, in general, have complications due to dual reuptake inhibition of 5-HT and NE when they are exposed late in the third trimester. In animal studies, levomilnacipran ER was not teratogenic during the period of organogenesis. However, a decrease in fetal weight was observed [45].

Discussion

The prevalence of depression has increased in recent years due to increased awareness about the depression. Despite the availability of several effective antidepressants, only 30% of patients take psychotropic medications as prescribed due to the presence of AEs, an absence of noticeable therapeutic effects for several weeks, and lack of response with the traditional antidepressants in some patients [5]. The first-line treatment of MDD includes administration of an SSRI, SNRIs, bupropion, and mirtazapine. However, the studies indicate that remission rates following two trials of an SSRI are less than 50%. This is particularly important considering the monoamine theory of depression, suggesting the role of serotonin, norepinephrine, and/or dopamine. This article reviews relevant articles to provide a comprehensive overview of some novel antidepressants including desvenlafaxine, vortioxetine, vilazodone, and levomilnacipran ER.

Vilazodone is a 5HT_1A_ partial agonistic and a serotonin transporter blockade. It is extensively metabolized mainly through CYP3A4 and should be taken with food. One study showed that patients taking Vilazodone did not report any sexual side effects as compared to placebo or citalopram group. Levomilnacipran, is another FDA approved new antidepressants, which acts as an SNRI but preferentially inhibit norepinephrine re-uptake as compared to other SNRIs.

Desvenlafaxine is an SNRI, with a higher affinity for serotonin transporter as compared to NE transporter. Along with an improvement in mood, it also results in improvement in the quality of life. The side effects and withdrawal symptoms need to be observed especially with higher doses. Vortioxetine works through various serotonin receptors and increases other neurotransmitters which help improvement in mood and cognition. It is metabolized through the liver and acts as a substrate mainly for CYP2D6. Because of its action on different receptors, there is a possibility of more side effects and withdrawal symptoms, which can lead to non-compliance with the treatment. 

## Conclusions

The multimodal mechanisms of actions of new antidepressants explain that depression may not be caused by the simple deficit of serotonin, but rather can be related to “flooding” 5HT_1A_ autoreceptors in midbrain peri-raphe areas by serotonin itself through the action of glutamate, noradrenaline, and histamine. There is limited research on the use of the novel antidepressants in human subjects, and further studies are warranted to reveal substantial difference and other novel attributes of these new medications.
